# Association of estrogen receptor single nucleotide polymorphisms and perinatal depression

**DOI:** 10.1371/journal.pone.0334705

**Published:** 2025-10-16

**Authors:** Richelle Duque Björvang, Lulu Francis Gumbo, Anders Årdahl, Susanne Lager, Erika Comasco, Emma Fransson, Alkistis Skalkidou

**Affiliations:** 1 Department of Women’s and Children’s Health, Uppsala University, Uppsala, Sweden; 2 National Bioinformatics Infrastructure Sweden, Department of Cell and Molecular Biology, Uppsala University, Uppsala, Sweden; 3 Department of Women’s and Children’s Health, Science for Life Laboratory, Uppsala University, Uppsala, Sweden; National Institute of Child Health and Human Development (NICHD), NIH, UNITED STATES OF AMERICA

## Abstract

Depression during pregnancy and in the postpartum period have been receiving increasing attention considering the possible complications for the mother and baby if left untreated. Genetic variations in the estrogen receptor genes (*ESR*) have been implicated in susceptibility to depression. However, only few studies investigated them in perinatal depression (PND) and none on its different trajectories (i.e., patterns of time of onset and persistency of depression). Here, we explored the association of single nucleotide polymorphisms (SNPs) of the *ESR1* and *ESR2* genes with PND among 2,973 women in Sweden. PND was defined using the Edinburgh Postnatal Depression Scale, the Depression Self-Rating Scale, use of selective serotonin reuptake inhibitor, and/or medical records. PND trajectories were identified as follows: controls (no depression at any point in the perinatal period), antepartum (depression during pregnancy and resolved postpartum), postpartum-onset (no depression during pregnancy with onset after delivery), and persistent (depression throughout the perinatal period). Multivariable logistic regression was performed. Out of 56 SNPs analyzed, one SNP in the *ESR1* gene (rs2982712) was nominally significantly associated with PND (OR 0.83, 95% CI 0.71–0.98, p = 0.03) as well as with persistent depression (OR 0.77, 95% CI 0.61–0.98, p = 0.03) in the overdominant model (DD/dd vs. Dd). In addition, we also found two SNPs, namely rs1884051 (OR 0.74, 95% CI 0.56–0.98, p = 0.03) and rs2228480 (OR 0.77, 95% CI 0.60–0.99, p = 0.04) in the *ESR1* gene, that were nominally significantly associated with persistent depression only. None of the *ESR1* SNPs were associated with antepartum or postpartum-onset depression. None of the *ESR2* SNPs, nor any haplotypes, were associated with PND or its trajectories. Our findings suggest a role of *ESR1* in PND, especially its persistent trajectory.

## Introduction

Perinatal depression (PND) is the most common mental disorder associated with childbirth [1]. It is clinically defined as a major depressive disorder with perinatal onset; that is, during pregnancy and/or four weeks following child birth [2]. The duration following childbirth frequently extends up to one year in clinical and research settings [3]. The global prevalence of PND has been estimated to be approximately 17% [1]. PND poses biological, psychological and social complications, both to the mother and the baby, as well as the society [4,5]. It may be further categorized into three trajectories according to the onset and duration of symptoms, namely (1) antepartum depression with remission during postpartum, (2) postpartum-onset depression with no depressive symptoms during pregnancy, and (3) persistent depression occurring in both the antepartum and postpartum period with no remission [6].

While the pathophysiology of PND is not clearly understood, some studies have attributed sensitivity to fluctuation of gonadal hormones to the occurrence of depression during and after pregnancy [7–9]. The role of gonadal hormones in depression has also been suggested in other periods of the female´s reproductive life, from puberty to menopause [10]. Pregnant women experience an exponential increase in levels of estrogen and progesterone that sharply drop postpartum [7,11]. In the brain, estrogen has been shown to promote the synthesis of serotonin, inhibiting its degradation and reuptake, and increasing the expression of serotonin receptors [8,12]. Also, in the presence of progesterone, estrogen has been shown to increase the level of tryptophan, a precursor for serotonin [13]. Serotonin is a neurotransmitter involved in mood, sleep, sexual behavior, and cognitive function, and the target of many antidepressants, such as serotonin reuptake inhibitors, which are used to treat PND.

Estrogen receptors are expressed in several areas of the brain, especially the cerebral cortex and the limbic system, regions known to be involved in emotions, mood, and behavior [14]. Moreover, studies on DNA methylation [15,16] as well as mRNA expression [17–19] of genes related to the estrogen pathway further support the hypothesis of increased sensitivity to estrogen signaling in PND. More specifically, the *TTC9B* and *HP1BP3* genes have been identified in estradiol-associated epigenomic profiles to predict postpartum mood episodes [15,16]. In addition, 39 transcripts expressed during the third trimester involved in estrogen-signaling pathways were significantly enriched in postpartum-onset depression [17].

Hypotheses on estrogen involvement in the etiology of PND have extended into the realm of genetics, where single nucleotide polymorphisms (SNPs) on estrogen receptor genes (*ESR*) are implicated. Two types of nuclear estrogen receptors are estrogen receptor alpha (ERα) and estrogen receptor beta (ERβ). They are encoded by the gene *Estrogen Receptor 1 and 2 (ESR 1 and ESR 2)* located on chromosome 6q25.1 and 14q22-24, respectively. A meta-analysis of ten studies has shown the association of 4 *ESR* SNPs (rs2234693, rs9340799, rs4986938, rs1256049) with depression in women [20]. However, there are only few studies specifically investigating the association with PND [21–25] and there is none that examined ESR genotype in relation with trajectories of PND. Hence, this large, population-based study aimed to explore the association of SNPs of the *ESR1* and *ESR2* genes with PND and its trajectories. A secondary aim was to explore the role of estradiol levels in late pregnancy as a possible effect modifier of associations, in a subset of individuals.

## Materials and methods

### Study participants

This is a nested study within the population-based prospective Biology, Affect, Stress, Imaging, and Cognition (BASIC) cohort conducted from September 9^th^ 2009 to November 30^th^ 2019 in Uppsala, Sweden [26]. The main aim of BASIC was to investigate the biopsychosocial processes in PND and to identify risk factors to improve early detection. The BASIC study has been described in detail previously [26]. Briefly, all women attending routine prenatal ultrasound were invited to participate and signed written informed consent forms. Exclusion criteria were age below 18 years, inability to read and understand Swedish language, protected identity, known blood-borne infections and non-viable pregnancy as diagnosed by routine ultrasound. All consenting participants completed web-based questionnaires on sociodemographic variables as well as medical conditions including ongoing medications and prior psychiatric history. In the medical history section of the questionnaire, participants were asked whether they have had depression to which they answered either “Yes” or “No”. They were also asked if they were attended by someone for their mental health. Individuals were then followed-up at gestational week 32, and 6 weeks and 6 months postpartum. In addition, blood samples were collected in mid- and late pregnancy and during delivery. Blood samples were separated with centrifugation where the buffy coat and plasma were stored at −70°C. Written, informed consent was obtained from all participants in accordance with the Declaration of Helsinki. This study has been approved by the Regional Ethical Review Board in Uppsala (Dnr 2009/171 with amendments).

### Outcome- Perinatal Depression

In the current study, the outcome PND was determined using the Edinburg Postnatal Depression Scale (EPDS), Mini International Neuropsychiatric scale (MINI), Depression Self-Rating Scale (DSRS), use of selective serotonin reuptake inhibitor (SSRI) during pregnancy and/or postpartum, and/or diagnosis reported in national registers (ICD-10 codes F32 and F53). In this study, a cut-off point of EPDS ≥13 during pregnancy and EPDS ≥12 during postpartum were used to indicate depressive symptoms [27,28]. Individuals were classified as having PND if any of the assessments above have been fulfilled at any point during the perinatal period. In addition, PND was further categorized into trajectory groups based on onset and resolution/persistence of symptoms: controls (no depression during the perinatal period), antepartum depression (depression during pregnancy only and resolved postpartum), postpartum-onset depression (no depression during pregnancy and developed depression during postpartum), and persistent depression (depression throughout the perinatal period from pregnancy until postpartum).

### Genetic analyses

DNA was extracted from blood using the silica-based Kleargene XL Nucleic acid extraction kit. Genotyping for *ESR1* and *ESR2* was performed for 2,915 individuals genotyped using the Illumina Infinium assay and Illumina Global Screening Array – Multi Diseases version 2 (GSA-MDv2) at the SNP&SEQ Technology Platform, SciLifeLab, Uppsala University. One SNP of *ESR1* (rs1884051) was performed for 1,425 individuals using the Kbioscience Allele-Specific Polymorphism assay (KASP) based on competitive allele-specific PCR and bi-allelic scoring of the SNP (KBioscience/LGC®, UK). No-template control samples were included to enable the detection of contamination or non-specific amplification. Minor allele frequency was checked. Two SNPs with minor allele frequency below 0.05 were excluded (S1 Table). To detect genotype calling error, genotype frequencies were tested for Hardy-Weinberg equilibrium. One SNP with Hardy-Weinberg equilibrium p-value <0.05 was excluded. Linkage disequilibrium was measured with R^2^, where R^2^ = 0 indicated independent alleles while R^2^ = 1 means that an allele of one variant predicts the allele of another variant. Haplotype blocks were estimated using the expectation maximization algorithm.

### Estradiol analysis

Among the BASIC participants who donated blood in late pregnancy, estradiol was analyzed in a subset of individuals (N = 205). This subset was oversampled for cases with ongoing depression (i.e., EPDS ≥13 at either gestational week 17 or 32 and/or taking SSRI during pregnancy). Competitive immunometry electrochemiluminescense was used to detect estradiol in serum. Analyses were performed with a Roche Cobas Elecsys estradiol III kit (Roche Diagnostics, Bromma, Sweden). Total coefficient of variation was 11% at 110 pmol/L and 3% at 2,000 pmol/L, analysis.

### Statistical analyses

Differences in characteristics across trajectories were tested with T-test, Wilcoxon Rank Sum Test, Chi-square test or Fisher’s Exact test, as appropriate. Shapiro-wilk test was done to test normality of the distribution. To determine which specific trajectories differed, post-hoc pairwise comparisons were performed for those characteristics significantly associated with PND trajectories. Post-hoc pairwise comparisons p-values were adjusted with false discovery rate, thereby, called q-values. Individuals with missing data on PND, or depression history were excluded from further analyses. Individuals with missing data on each SNP marker were excluded from analysis only for that specific SNP. For the genetic analyses, logistic regression was utilized to evaluate the association of SNPs with PND. The outcomes of interest were (1) PND vs. no PND, and (2) trajectories of PND with controls as reference. Several genotype models were explored: codominant (DD vs. Dd vs. dd), dominant (DD vs. Dd/dd), recessive (DD/Dd vs. dd), overdominant (DD/dd vs. Dd), and additive (DD < Dd < dd). Models were adjusted for depression history, pre-pregnancy BMI and education. Linkage disequilibrium were estimated for the SNPs in each gene. For the SNPs with high linkage disequilibrium, logistic regression between the outcome and haplotypes with at least 5% frequency was performed. Odds ratios (ORs) were obtained for each haplotype with the most common haplotype as the reference. Moreover, a sub-analysis was performed on a subset of individuals (N = 205). To explore effect modification of estradiol levels in late pregnancy on the SNPs that were nominally significant associated with PND and/or its trajectories, an interaction term between estradiol (categorized as “below the median” and “above the median”) and each SNP was added to the model. However, the postpartum-onset depression trajectory was excluded because of low sample size in this subset (N = 5). All statistical analyses were performed using R (version 4.3.1) and RStudio (version 2023.12.1 Build 402), utilizing the packages SNPassoc [29], LDlinkR [30], and haplo.stats [31]. Significance level was set at p < 0.05 and Bonferroni correction for multiple testing was performed.

## Results

### Sample characteristics

Out of 2,973 individuals in this cohort, 268 (9%) reported antepartum depression, 403 (13.6%) had postpartum-onset depression and 417 (14%) had persistent depression. Cohort characteristics are found in Table 1. Among individuals with antepartum depression, there was a lower percentage who had university education (q < 0.001) and higher percentage who had history of depression (q < 0.001) compared to controls. Similar results were found when compared to postpartum-onset depression (q = 0.006 and q = 0.029, respectively). There was also a lower percentage who had history of depression (q < 0.001) compared to those with persistent depression. Among those with postpartum-onset depression, there was a higher percentage who had history of depression compared to controls (q < 0.001). There was also a higher percentage who had university education (q = 0.005) and lower percentage who had history of depression (q < 0.001) compared to those with persistent depression. Among individuals with persistent depression, there was higher pre-pregnancy body mass index (BMI) (q = 0.002), lower percentage who had university education (q < 0.001) and higher percentage who had history of depression (q < 0.001) compared to controls. Late pregnancy estradiol levels were not significantly different among the trajectories after correcting for post-hoc pairwise comparisons.

**Table 1:** Cohort characteristics.

		Controls (N = 1,885)	PND (N = 1,088)			p-value^a^
			Antepartum depression (N = 268)	Postpartum-onset depression (N = 403)	Persistent depression (N = 417)	
Age, years	Median [Min, Max]	31.0 [18.0, 48.0]	31.0 [19.0, 45.0]	31.0 [20.0, 45.0]	31.0 [19.0, 44.0]	0.15
Pre-pregnancy BMI, kg/m^2^	Median [Min, Max]	22.8 [15.8, 49.0]	23.5 [17.6, 43.7]	23.1 [17.0, 45.2]	23.5 [17.0, 50.9]	<0.001
	Missing	5 (0.3%)	3 (1.1%)	4 (1.0%)	2 (0.5%)	
Parity	Nulliparous	1001 (53.1%)	133 (49.6%)	224 (55.6%)	193 (46.3%)	0.07
	Primi/Multiparous	868 (46.0%)	131 (48.9%)	175 (43.4%)	211 (50.6%)	
	Missing	16 (0.8%)	4 (1.5%)	4 (1.0%)	13 (3.1%)	
Education	University	1434 (76.1%)	180 (67.2%)	304 (75.4%)	284 (68.1%)	<0.001
	Else	374 (19.8%)	81 (30.2%)	80 (19.9%)	124 (29.7%)	
	Missing	77 (4.1%)	7 (2.6%)	19 (4.7%)	9 (2.2%)	
Infant sex	Boy	867 (46.0%)	103 (38.4%)	175 (43.4%)	178 (42.7%)	0.08
	Girl	779 (41.3%)	133 (49.6%)	157 (39.0%)	169 (40.5%)	
	Missing	239 (12.7%)	32 (11.9%)	71 (17.6%)	70 (16.8%)	
History of depression/ Previous contact with psychiatrist	Yes	785 (41.6%)	205 (76.5%)	276 (68.5%)	376 (90.2%)	<0.001
	No	1100 (58.4%)	63 (23.5%)	127 (31.5%)	41 (9.8%)	
Total Estradiol (pM)^**b**^	Median [Min, Max]	79400 [9730, 299000]	87000 [38800, 179000]	110000 [69900, 161000]	90300 [20100, 153000]	0.04
	Missing	1818 (96.4%)	206 (76.9%)	398 (98.8%)	346 (83.0%)	

^a^ANOVA, Kruskal-wallis test, Chi-square test, or Fishers exact test, as appropriate. Shapiro-wilk test done to determine normality of distribution

^b^Available for only N = 205 individuals (controls N = 67, antepartum depression N = 62, postpartum-onset depression N = 5, persistent depression N = 71)

### *ESR* SNPs, PND and its trajectories

**Fig 1:**
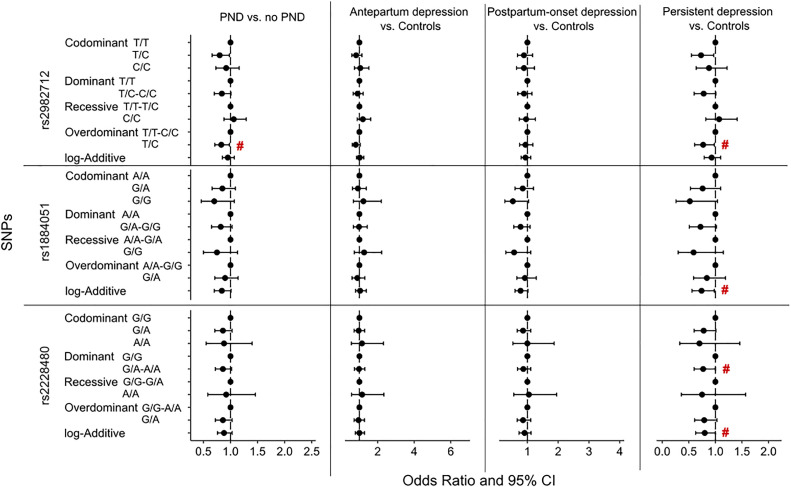
Forest plot of odds ratio (OR) and confidence intervals (95% CI) of the association of *ESR* SNPs to PND and its trajectories. Logistic regression adjusted for depression history, pre-pregnancy BMI and education. # p < 0.05.

The genotype frequencies are shown in the supplementary S2 Table. Three SNPs in *ESR1* (rs1884051, rs2982712, and rs2228480) were nominally significantly associated with PND or its trajectories in at least one genotype model (Fig 1). No associations were significant among the *ESR2* SNPs*.* In the overdominant model of rs2982712, there was 17% (OR 0.83, 95% CI 0.71–0.98, p = 0.03) lower odds of PND in heterozygous individuals compared to those with homozygous genotypes. When investigating the trajectories of PND, similar results were seen in the overdominant model of rs2982712 among those with persistent depression compared to controls (OR 0.77, 95% CI 0.61–0.98, p = 0.03) (Fig 1). In addition, there were SNPs that were nominally significant only in the persistent depression group compared to controls, but not in the general group of PND. In the log-additive model for rs1884051, individuals with heterozygous genotype had 26% (OR 0.74, 95% CI 0.56–0.98, p = 0.03) lower odds for persistent depression compared to those with homozygous for the G allele (Fig 1). In the dominant model for rs2228480, those carrying the A allele had 23% (OR 0.77, 95% CI 0.60–0.99, p = 0.04) lower odds than those with G/G genotype (Fig 1). No SNP was significantly associated with antepartum depression, or postpartum-onset depression. These associations did not survive correction for multiple testing.

**Fig 2:**
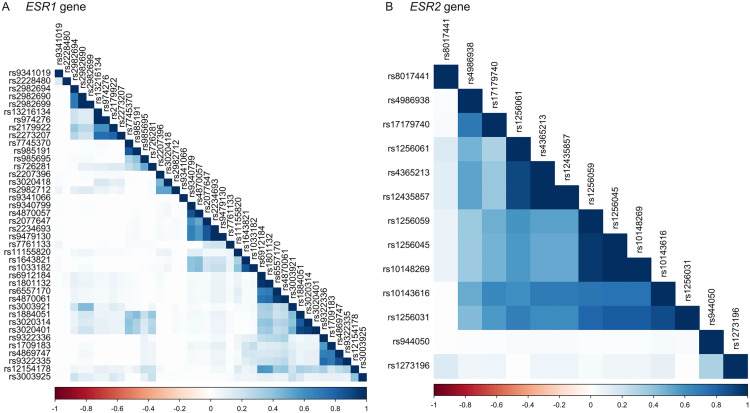
Linkage disequilibrium plots of SNPs in *ESR1* and *ESR2* genes. **(A)** *ESR1* and **(B)** *ESR2* linkage disequilibrium plots where red signifies and r^2^ = −1 while blue denotes r^2^ = 1.

*ESR1* showed low linkage disequilibrium (Fig 2A). Hence, no haplotype in *ESR1* was estimated. On the other hand, *ESR2* showed high linkage disequilibrium (Fig 2B). Haplotypes in *ESR2* were estimated, where their association with PND and its trajectories were examined. None of the *ESR2* gene haplotypes were significantly associated with PND nor its trajectories.

For the three SNPs that were nominally significantly associated with PND and/or its trajectories, possible effect modification of estradiol levels in late pregnancy on the SNP was further explored in a subset of 205 individuals. The interaction term between estradiol and each SNP was not significantly associated with PND nor its trajectories in any of the genetic models.

## Discussion

This study explored the association of *ESR* SNPs with PND, including its trajectories. We found one SNP in the *ESR1* gene (rs2982712) that was nominally significantly associated to PND in general, as well as the persistent trajectory. In addition, we also found two SNPs (rs1884051 and rs2228480 in *ESR1* gene) that were nominally significantly associated specifically with persistent depression. To the best of our knowledge, these SNPs have not been previously reported and may be potential loci of relevance to PND.

In our study, one SNP (rs2982712 in the *ESR1* gene) was nominally significantly associated with PND, while three SNPs (rs1884051, rs2982712, and rs2228480 in *ESR1* gene) were associated with persistent depression. It is interesting to note that two additional SNPs were found in the persistent depression group that were not significant when considering PND as one entity. This supports studies suggesting the different trajectories being distinct in terms of biological and psychosocial vulnerability [6]. So far, it has been shown that younger age, unemployment and lower educational attainment were associated with antepartum depression and persistent depression while pregnancy complications, nulliparity and negative delivery experiences were associated with postpartum-onset depression [6]. Further, specific biological differences have been noted between the different trajectories, with examples including higher CRH levels in mid-pregnancy and evening cortisol postpartum among do novo postpartum depressed, different metabolomics profiles among persistent and de novo depressed, as well as differences in inflammatory markers and telomere length [32–36]. Our results are in line and add to this growing literature. To the best of our knowledge, there are no studies on the relationship of rs2982712 with PND or with persistent depression through the perinatal period. Our finding on rs1884051 was in line with Pinsonneault et al. (2013), where they did not find any association with PND [22]. Similarly, Tan et al. (2018) did not find any association of rs2228480 with PND [21]. None of the these three SNPs were found to be significant in meta-analyses of genome-wide association studies for postpartum depression [37] or major depression [38].

Previous studies have suggested functional implications for the three identified SNPs. For instance, women carrying the minor genotype for rs2982712 exhibit lower 17β-estradiol levels throughout the menstrual cycle [39]. Additionally, the minor genotype for rs1884051 has been associated with elevated free estradiol levels in women with hyperandrogenism [40]. These findings indicate potential biological significance of these variants, warranting further investigation into their role in perinatal depression.

Earlier studies reported conflicting results regarding the association of *ESR* SNPs and PND. While an association with postpartum depression was found for rs2234693, rs2077647, rs3020434, rs9340954, rs19340958, and rs3020363 in some studies [22,23,25], others did not find an association for rs2234693, rs2077647, rs9340799, and rs488133 [21,24]. In addition, a meta-analysis of genome-wide association studies for postpartum depression did not find any significant SNPs in the European ancestry [37]. For all the SNPs mentioned, we did not find any association in our study. One possible explanation could be the difference in definitions of PND. Different measures such as interviews, self-reports, medical records and register data were used to define PND. Moreover, previous studies focused mainly on the postpartum period, not considering the time of onset, if this was, for example, already present during pregnancy and there was persistence of symptoms.

Previous studies have shown several SNPs to be associated with major depression in women. In a meta-analysis of 10 studies by Li *et al.* (2022), four SNPs, specifically rs2234693 (Pvull), rs9340799 (Xbal), rs4986938, rs1256049, were associated with depression. None of these SNPs were significant in our study. However, seven out of the ten studies were performed in Asians, while only other three were in Caucasians. In addition, most of the studies were focused on major depression or perimenopausal depression. This could explain the possible lack of significance in our study. It remains inconclusive whether PND and major depression with an onset outside the perinatal period have similar genetic make-up. While some studies have shown similar genetic underpinnings [41,42], other have demonstrated PND and major depression to be distinct genetic disorders [43,44].

The strengths of the present study include the population-based longitudinal design, which allowed investigation of the trajectories of PND, addressing its heterogenous nature, by forming groups based on onset and remission/persistence of symptoms. Moreover, several genetic models have been considered in this paper. The current study is also one of the largest in the field, including 2,973 individuals. Moreover, the rich data collected from the individuals through questionnaires and interviews as well as linking diagnoses with registers led to an inclusive characterization of PND. However, we also acknowledge the limitations of this study. Ethnicity was last recorded in the Swedish national registers in 1945 [45]; hence, such data were not available. Moreover, we had only data on 205 individuals with estradiol levels. Hence, the lack of statistical significance taking into consideration the estradiol levels might be due to the low power. These factors limit the generalizability of our findings. Further research is warranted to validate these results in diverse populations. Moreover, all the significant associations seen were nominal. Hence, results need to be interpreted cautiously and call for independent replication. In addition, given the growing literature on distinct characteristics of the different trajectories, future studies could consider classification of PPD cases in these trajectories, in order to further facilitate their full characterization and to promote a better understanding of the different biological and psychosocial processes that shape the risk for peripartum depression.

## Conclusions

Three estrogen receptor-related SNPs were found to be associated with PND and/or its trajectories, based on symptom onset and persistence. Genetic variants in the estrogen receptor may play a role in PND, especially when symptoms persist throughout pregnancy into the postpartum period.

## Supporting information

S1 Table Genotype information for all SNPs analysed. (DOCX)

S2 Table Genotype frequencies across PND trajectory groups. (DOCX)
