# Supplementary material for: Association of estrogen receptor single nucleotide polymorphisms and perinatal depression
Source: PLoS One. 2025 Oct 16;20(10):e0334705. doi: 10.1371/journal.pone.0334705 (PMC12530586; doi:10.1371/journal.pone.0334705)
Supplement: S1 Table — (DOCX) [file pone.0334705.s001.docx]

Supplementary Table 1. Genotype information for all SNPs analysed. Highlighted cells are basis for exclusion (minor allele frequency <0.05 or Hardy-Weinberg Equilibrium p-value<0.05) of SNP markers.

| **Marker** | **Chr** | **Position** | **Gene** | **Call rate (%)** | **Minor**  **allele** | **Major**  **allele** | **Minor**  **Allele**  **Frequency (%)** | **Major**  **allele**  **frequency (%)** | **Hardy-Weinberg**  **Equilibrium**  **p-value** | **Missing**  **(%)** |
| --- | --- | --- | --- | --- | --- | --- | --- | --- | --- | --- |
| rs2077647 | 6 | 152129077 | *ESR1* | 99,9 | T | C | 45.7 | 54.3 | 0.89 | 1.7 |
| rs7761133 | 6 | 152151863 | *ESR1* | 99,8 | T | C | 15.6 | 84.4 | 0.86 | 1.9 |
| rs2234693 | 6 | 152163335 | *ESR1* | 99,8 | T | C | 43.3 | 56.7 | 0.48 | 1.9 |
| rs9340799 | 6 | 152163381 | *ESR1* | 100,0 | A | G | 30.6 | 69.4 | 0.78 | 1.7 |
| rs9479130 | 6 | 152168456 | *ESR1* | 99,9 | A | C | 43.1 | 56.9 | 0.51 | 1.8 |
| rs4870057 | 6 | 152171898 | *ESR1* | 99,9 | A | G | 29.3 | 70.7 | 0.69 | 1.7 |
| rs1643821 | 6 | 152183551 | *ESR1* | 99,9 | G | A | 38.0 | 62.0 | 0.96 | 1.7 |
| rs1709183 | 6 | 152193996 | *ESR1* | 99,9 | T | C | 29.1 | 70.9 | 0.17 | 1.7 |
| rs1033182 | 6 | 152195034 | *ESR1* | 100,0 | G | A | 34.4 | 65.6 | 0.41 | 1.7 |
| rs4869747 | 6 | 152198111 | *ESR1* | 99,8 | A | C | 23.9 | 76.1 | 0.95 | 1.7 |
| rs9322335 | 6 | 152200129 | *ESR1* | 100,0 | C | T | 25.5 | 74.5 | 0.36 | 1.7 |
| rs9322336 | 6 | 152200430 | *ESR1* | 99,9 | T | C | 21.0 | 79.0 | 0.57 | 1.7 |
| rs6557170 | 6 | 152203104 | *ESR1* | 99,9 | G | A | 23.3 | 76.7 | 0.51 | 1.7 |
| rs11155820 | 6 | 152204210 | *ESR1* | 100,0 | A | G | 30.7 | 69.3 | 0.13 | 1.7 |
| rs4870061 | 6 | 152237468 | *ESR1* | 99,9 | C | T | 23.6 | 76.4 | 0.56 | 1.8 |
| rs12154178 | 6 | 152251080 | *ESR1* | 99,8 | A | C | 30.0 | 70.0 | 0.58 | 1.9 |
| rs6912184 | 6 | 152260206 | *ESR1* | 99,9 | A | G | 22.8 | 77.2 | 0.38 | 1.7 |
| rs1801132 | 6 | 152265522 | *ESR1* | 99,8 | C | G | 22.8 | 77.2 | 0.38 | 1.8 |
| rs3020314 | 6 | 152270672 | *ESR1* | 100,0 | T | C | 33.0 | 67.0 | 0.49 | 1.7 |
| rs7745370 | 6 | 152274260 | *ESR1* | 99,9 | T | C | 11.4 | 88.6 | 0.29 | 1.8 |
| rs3003921 | 6 | 152279514 | *ESR1* | 99,8 | C | T | 20.8 | 79.2 | 0.89 | 1.8 |
| rs3020401 | 6 | 152283044 | *ESR1* | 100,0 | A | G | 31.1 | 68.9 | 0.87 | 1.7 |
| rs1884051 | 6 | 152283279 | *ESR1* | NA | A | G | 30.3 | 69.7 | 0.82 | 52.1* |
| rs985191 | 6 | 152283458 | *ESR1* | 99,9 | A | C | 10.5 | 89.5 | 0.62 | 1.7 |
| rs3003925 | 6 | 152284458 | *ESR1* | 99,9 | A | G | 20.5 | 79.5 | 0.31 | 1.8 |
| rs2982690 | 6 | 152285491 | *ESR1* | 99,9 | A | G | 18.6 | 81.4 | 0.64 | 1.8 |
| rs2982694 | 6 | 152285687 | *ESR1* | 99,9 | T | G | 16.3 | 83.7 | 0.30 | 1.9 |
| rs2982699 | 6 | 152286130 | *ESR1* | 100,0 | G | A | 18.7 | 81.3 | 0.59 | 1.7 |
| rs985695 | 6 | 152286705 | *ESR1* | 99,9 | C | T | 15.3 | 84.7 | 0.72 | 1.8 |
| rs2179922 | 6 | 152297100 | *ESR1* | 99,7 | G | A | 11.3 | 88.7 | 0.42 | 2.0 |
| rs726281 | 6 | 152302578 | *ESR1* | 100,0 | A | G | 28.0 | 72.0 | 0.09 | 1.7 |
| rs13216134 | 6 | 152328484 | *ESR1* | 100,0 | A | G | 13.3 | 86.7 | 0.69 | 1.7 |
| rs3020418 | 6 | 152345162 | *ESR1* | 99,9 | G | A | 32.0 | 68.0 | 0.92 | 1.8 |
| rs2982712 | 6 | 152358179 | *ESR1* | 99,9 | T | C | 48.6 | 51.4 | 0.35 | 1.7 |
| rs2273207 | 6 | 152382325 | *ESR1* | 100,0 | A | G | 11.1 | 88.9 | 0.91 | 1.7 |
| rs2207396 | 6 | 152382382 | *ESR1* | 100,0 | G | A | 24.3 | 75.7 | 0.49 | 1.7 |
| rs974276 | 6 | 152382420 | *ESR1* | 99,8 | A | G | 13.8 | 86.2 | 0.63 | 1.9 |
| rs9341019 | 6 | 152382688 | *ESR1* | 100,0 | A | C | 7.6 | 92.4 | 0.14 | 1.7 |
| rs9341066 | 6 | 152419526 | *ESR1* | 99,9 | G | A | 5.0 | 95.0 | 0.61 | 9.6 |
| rs2228480 | 6 | 152420095 | *ESR1* | 100,0 | G | A | 18.4 | 81.6 | 0.94 | 1.7 |
| rs4986938 | 14 | 64699816 | *ESR2* | 99,9 | C | T | 35.4 | 64.6 | 0.92 | 1.8 |
| rs944050 | 14 | 64700045 | *ESR2* | 100,0 | T | C | 5.1 | 94.9 | 0.63 | 9.5 |
| rs1256061 | 14 | 64703593 | *ESR2* | 99,8 | G | T | 47.8 | 52.2 | 0.46 | 1.8 |
| rs1256059 | 14 | 64710417 | *ESR2* | 99,8 | G | A | 46.0 | 54.0 | 0.42 | 1.9 |
| rs8017441 | 14 | 64715794 | *ESR2* | 100,0 | A | G | 5.7 | 94.3 | 0.66 | 1.7 |
| rs4365213 | 14 | 64720264 | *ESR2* | 99,8 | T | C | 43.6 | 56.4 | 0.19 | 1.9 |
| rs12435857 | 14 | 64723525 | *ESR2* | 99,9 | G | A | 43.5 | 56.5 | 0.20 | 1.7 |
| rs1256045 | 14 | 64729760 | *ESR2* | 100,0 | C | A | 45.9 | 54.1 | 0.61 | 2.0 |
| rs10148269 | 14 | 64736924 | *ESR2* | 99,9 | G | A | 45.7 | 54.3 | 0.51 | 2.3 |
| rs1273196 | 14 | 64739505 | *ESR2* | 99,7 | A | G | 6.6 | 93.4 | 0.44 | 9.5 |
| rs10143616 | 14 | 64740175 | *ESR2* | 100,0 | G | A | 47.9 | 52.1 | 0.08 | 1.8 |
| rs1256031 | 14 | 64746179 | *ESR2* | 99,9 | A | G | 47.9 | 52.1 | 0.26 | 1.7 |
| rs17179740 | 14 | 64756751 | *ESR2* | 100,0 | G | A | 37.5 | 62.5 | 0.92 | 1.7 |
| **Excluded** | | | | | | | | | | |
| rs1256049 | 14 | 64724051 | *ESR2* | 99,9 | C | T | 4.9 | 95.1 | 0.62 | 9.5 |
| rs1256034 | 14 | 64745125 | *ESR2* | 99,9 | C | T | 4.9 | 95.1 | 0.80 | 9.5 |
| rs1952586 | 14 | 64759419 | *ESR2* | 99,9 | T | C | 10.7 | 89.3 | 0.02 | 1.8 |

*rs1884051 was performed for only 1,425 individuals using the Kbioscience Allele-Specific Polymorphism assay (KASP) based on competitive allele-specific PCR and bi-allelic scoring of the SNP.
