# Supplementary material for: Association of estrogen receptor single nucleotide polymorphisms and perinatal depression
Source: PLoS One. 2025 Oct 16;20(10):e0334705. doi: 10.1371/journal.pone.0334705 (PMC12530586; doi:10.1371/journal.pone.0334705)
Supplement: S2 Table — (DOCX) [file pone.0334705.s002.docx]

Supplementary Table 2. Genotype frequencies across PND trajectory groups

|  | **Controls** (N=1,885) | **Perinatal depression** (N=1,088) | | | **P-value** | **Total** (N=2,973) |
| --- | --- | --- | --- | --- | --- | --- |
|  |  | **Pregnancy depression** (N=268) | **Postpartum- onset depression** (N=403) | **Persistent**  **depression** (N=417) |  |  |
| **rs2077647** |  |  |  |  |  |  |
| CC | 382 (20.6%) | 64 (24.2%) | 83 (20.9%) | 87 (21.2%) | 0.679 | 616 (21.1%) |
| TC | 914 (49.4%) | 128 (48.5%) | 205 (51.6%) | 194 (47.2%) |  | 1441 (49.3%) |
| TT | 554 (29.9%) | 72 (27.3%) | 109 (27.5%) | 130 (31.6%) |  | 865 (29.6%) |
| **rs7761133** |  |  |  |  |  |  |
| CC | 46 (2.5%) | 9 (3.4%) | 8 (2.0%) | 11 (2.7%) | 0.665 | 74 (2.5%) |
| TC | 485 (26.3%) | 78 (29.8%) | 98 (24.7%) | 103 (25.1%) |  | 764 (26.2%) |
| TT | 1316 (71.3%) | 175 (66.8%) | 291 (73.3%) | 297 (72.3%) |  | 2079 (71.3%) |
| **rs2234693** |  |  |  |  |  |  |
| CC | 353 (19.1%) | 51 (19.3%) | 67 (16.9%) | 76 (18.5%) | 0.618 | 547 (18.8%) |
| TC | 891 (48.3%) | 131 (49.6%) | 212 (53.5%) | 196 (47.7%) |  | 1430 (49.0%) |
| TT | 601 (32.6%) | 82 (31.1%) | 117 (29.5%) | 139 (33.8%) |  | 939 (32.2%) |
| **rs9340799** |  |  |  |  |  |  |
| AA | 893 (48.3%) | 133 (50.4%) | 180 (45.3%) | 195 (47.4%) | 0.552 | 1401 (47.9%) |
| AG | 789 (42.6%) | 101 (38.3%) | 183 (46.1%) | 179 (43.6%) |  | 1252 (42.8%) |
| GG | 168 (9.1%) | 30 (11.4%) | 34 (8.6%) | 37 (9.0%) |  | 269 (9.2%) |
| **rs9479130** |  |  |  |  |  |  |
| AA | 604 (32.7%) | 82 (31.1%) | 119 (30.0%) | 141 (34.3%) | 0.643 | 946 (32.4%) |
| AC | 892 (48.3%) | 130 (49.2%) | 211 (53.1%) | 194 (47.2%) |  | 1427 (48.9%) |
| CC | 351 (19.0%) | 52 (19.7%) | 67 (16.9%) | 76 (18.5%) |  | 546 (18.7%) |
| **rs4870057** |  |  |  |  |  |  |
| AA | 928 (50.2%) | 138 (52.3%) | 185 (46.6%) | 205 (49.9%) | 0.232 | 1456 (49.8%) |
| AG | 770 (41.6%) | 97 (36.7%) | 184 (46.3%) | 170 (41.4%) |  | 1221 (41.8%) |
| GG | 152 (8.2%) | 29 (11.0%) | 28 (7.1%) | 36 (8.8%) |  | 245 (8.4%) |
| **rs1643821** |  |  |  |  |  |  |
| AA | 265 (14.3%) | 48 (18.2%) | 58 (14.6%) | 56 (13.6%) | 0.492 | 427 (14.6%) |
| GA | 869 (47.0%) | 110 (41.7%) | 195 (49.1%) | 195 (47.4%) |  | 1369 (46.9%) |
| GG | 716 (38.7%) | 106 (40.2%) | 144 (36.3%) | 160 (38.9%) |  | 1126 (38.5%) |
| **rs1709183** |  |  |  |  |  |  |
| CC | 167 (9.0%) | 23 (8.7%) | 34 (8.6%) | 40 (9.7%) | 0.986 | 264 (9.0%) |
| TC | 736 (39.8%) | 112 (42.4%) | 161 (40.6%) | 164 (39.9%) |  | 1173 (40.1%) |
| TT | 947 (51.2%) | 129 (48.9%) | 202 (50.9%) | 207 (50.4%) |  | 1485 (50.8%) |
| **rs1033182** |  |  |  |  |  |  |
| AA | 225 (12.2%) | 38 (14.4%) | 48 (12.1%) | 47 (11.4%) | 0.573 | 358 (12.3%) |
| GA | 817 (44.2%) | 105 (39.8%) | 189 (47.6%) | 180 (43.8%) |  | 1291 (44.2%) |
| GG | 807 (43.6%) | 121 (45.8%) | 160 (40.3%) | 184 (44.8%) |  | 1272 (43.5%) |
| **rs4869747** |  |  |  |  |  |  |
| AA | 1073 (58.0%) | 151 (57.4%) | 238 (59.9%) | 240 (58.4%) | 0.908 | 1702 (58.3%) |
| AC | 671 (36.3%) | 97 (36.9%) | 133 (33.5%) | 143 (34.8%) |  | 1044 (35.7%) |
| CC | 106 (5.7%) | 15 (5.7%) | 26 (6.5%) | 28 (6.8%) |  | 175 (6.0%) |
| **rs9322335** |  |  |  |  |  |  |
| CC | 1042 (56.3%) | 143 (54.2%) | 225 (56.7%) | 230 (56.0%) | 0.944 | 1640 (56.1%) |
| CT | 683 (36.9%) | 103 (39.0%) | 142 (35.8%) | 148 (36.0%) |  | 1076 (36.8%) |
| TT | 125 (6.8%) | 18 (6.8%) | 30 (7.6%) | 33 (8.0%) |  | 206 (7.1%) |
| **rs9322336** |  |  |  |  |  |  |
| CC | 81 (4.4%) | 16 (6.1%) | 19 (4.8%) | 24 (5.8%) | 0.77 | 140 (4.8%) |
| TC | 592 (32.0%) | 88 (33.3%) | 129 (32.5%) | 136 (33.1%) |  | 945 (32.4%) |
| TT | 1176 (63.6%) | 160 (60.6%) | 249 (62.7%) | 251 (61.1%) |  | 1836 (62.9%) |
| **rs6557170** |  |  |  |  |  |  |
| AA | 94 (5.1%) | 10 (3.8%) | 23 (5.8%) | 23 (5.6%) | 0.863 | 150 (5.1%) |
| GA | 669 (36.2%) | 104 (39.4%) | 144 (36.4%) | 144 (35.0%) |  | 1061 (36.3%) |
| GG | 1087 (58.8%) | 150 (56.8%) | 229 (57.8%) | 244 (59.4%) |  | 1710 (58.5%) |
| **rs11155820** |  |  |  |  |  |  |
| AA | 902 (48.8%) | 136 (51.5%) | 181 (45.6%) | 201 (48.9%) | 0.736 | 1420 (48.6%) |
| AG | 759 (41.0%) | 101 (38.3%) | 179 (45.1%) | 171 (41.6%) |  | 1210 (41.4%) |
| GG | 189 (10.2%) | 27 (10.2%) | 37 (9.3%) | 39 (9.5%) |  | 292 (10.0%) |
| **rs4870061** |  |  |  |  |  |  |
| CC | 1077 (58.3%) | 151 (57.2%) | 224 (56.4%) | 240 (58.4%) | 0.916 | 1692 (57.9%) |
| CT | 674 (36.5%) | 103 (39.0%) | 150 (37.8%) | 148 (36.0%) |  | 1075 (36.8%) |
| TT | 97 (5.2%) | 10 (3.8%) | 23 (5.8%) | 23 (5.6%) |  | 153 (5.2%) |
| **rs12154178** |  |  |  |  |  |  |
| AA | 919 (49.8%) | 121 (45.8%) | 191 (48.2%) | 199 (48.4%) | 0.908 | 1430 (49.0%) |
| AC | 760 (41.2%) | 116 (43.9%) | 171 (43.2%) | 176 (42.8%) |  | 1223 (41.9%) |
| CC | 167 (9.0%) | 27 (10.2%) | 34 (8.6%) | 36 (8.8%) |  | 264 (9.1%) |
| **rs6912184** |  |  |  |  |  |  |
| AA | 1106 (59.8%) | 155 (58.7%) | 223 (56.2%) | 244 (59.4%) | 0.921 | 1728 (59.1%) |
| AG | 658 (35.6%) | 98 (37.1%) | 154 (38.8%) | 148 (36.0%) |  | 1058 (36.2%) |
| GG | 86 (4.6%) | 11 (4.2%) | 20 (5.0%) | 19 (4.6%) |  | 136 (4.7%) |
| **rs1884051** |  |  |  |  |  |  |
| AA | 434 (47.0%) | 64 (46.7%) | 97 (54.5%) | 101 (54.3%) | 0.109 | 696 (48.8%) |
| GA | 396 (42.9%) | 55 (40.1%) | 70 (39.3%) | 73 (39.2%) |  | 594 (41.7%) |
| GG | 94 (10.2%) | 18 (13.1%) | 11 (6.2%) | 12 (6.5%) |  | 135 (9.5%) |
| **rs1801132** |  |  |  |  |  |  |
| CC | 1103 (59.8%) | 156 (59.1%) | 224 (56.4%) | 243 (59.1%) | 0.951 | 1726 (59.2%) |
| CG | 657 (35.6%) | 97 (36.7%) | 153 (38.5%) | 149 (36.3%) |  | 1056 (36.2%) |
| GG | 86 (4.7%) | 11 (4.2%) | 20 (5.0%) | 19 (4.6%) |  | 136 (4.7%) |
| **rs3020314** |  |  |  |  |  |  |
| CC | 205 (11.1%) | 33 (12.5%) | 39 (9.8%) | 43 (10.5%) | 0.758 | 320 (11.0%) |
| TC | 801 (43.3%) | 117 (44.3%) | 179 (45.1%) | 194 (47.2%) |  | 1291 (44.2%) |
| TT | 844 (45.6%) | 114 (43.2%) | 179 (45.1%) | 174 (42.3%) |  | 1311 (44.9%) |
| **rs7745370** |  |  |  |  |  |  |
| CC | 28 (1.5%) | 5 (1.9%) | 8 (2.0%) | 5 (1.2%) | 0.842 | 46 (1.6%) |
| TC | 359 (19.4%) | 54 (20.5%) | 72 (18.1%) | 88 (21.4%) |  | 573 (19.6%) |
| TT | 1461 (79.1%) | 205 (77.7%) | 317 (79.8%) | 318 (77.4%) |  | 2301 (78.8%) |
| **rs3003921** |  |  |  |  |  |  |
| CC | 1159 (62.7%) | 161 (61.0%) | 248 (62.5%) | 251 (61.4%) | 0.917 | 1819 (62.3%) |
| CT | 611 (33.1%) | 94 (35.6%) | 135 (34.0%) | 144 (35.2%) |  | 984 (33.7%) |
| TT | 78 (4.2%) | 9 (3.4%) | 14 (3.5%) | 14 (3.4%) |  | 115 (3.9%) |
| **rs3020401** |  |  |  |  |  |  |
| AA | 881 (47.6%) | 122 (46.2%) | 190 (47.9%) | 191 (46.5%) | 0.99 | 1384 (47.4%) |
| AG | 789 (42.6%) | 114 (43.2%) | 171 (43.1%) | 182 (44.3%) |  | 1256 (43.0%) |
| GG | 180 (9.7%) | 28 (10.6%) | 36 (9.1%) | 38 (9.2%) |  | 282 (9.7%) |
| **rs985191** |  |  |  |  |  |  |
| AA | 1487 (80.4%) | 206 (78.0%) | 324 (81.6%) | 329 (80.0%) | 0.703 | 2346 (80.3%) |
| AC | 340 (18.4%) | 52 (19.7%) | 67 (16.9%) | 78 (19.0%) |  | 537 (18.4%) |
| CC | 22 (1.2%) | 6 (2.3%) | 6 (1.5%) | 4 (1.0%) |  | 38 (1.3%) |
| **rs3003925** |  |  |  |  |  |  |
| AA | 1181 (63.9%) | 160 (60.6%) | 248 (62.5%) | 260 (63.3%) | 0.729 | 1849 (63.3%) |
| AG | 584 (31.6%) | 95 (36.0%) | 134 (33.8%) | 130 (31.6%) |  | 943 (32.3%) |
| GG | 83 (4.5%) | 9 (3.4%) | 15 (3.8%) | 21 (5.1%) |  | 128 (4.4%) |
| **rs2982690** |  |  |  |  |  |  |
| AA | 1232 (66.6%) | 174 (65.9%) | 260 (65.5%) | 268 (65.4%) | 0.961 | 1934 (66.2%) |
| AG | 551 (29.8%) | 82 (31.1%) | 125 (31.5%) | 125 (30.5%) |  | 883 (30.2%) |
| GG | 66 (3.6%) | 8 (3.0%) | 12 (3.0%) | 17 (4.1%) |  | 103 (3.5%) |
| **rs2982694** |  |  |  |  |  |  |
| GG | 54 (2.9%) | 6 (2.3%) | 10 (2.5%) | 14 (3.4%) | 0.902 | 84 (2.9%) |
| TG | 486 (26.3%) | 74 (28.0%) | 115 (29.0%) | 109 (26.6%) |  | 784 (26.9%) |
| TT | 1306 (70.7%) | 184 (69.7%) | 271 (68.4%) | 287 (70.0%) |  | 2048 (70.2%) |
| **rs2982699** |  |  |  |  |  |  |
| AA | 67 (3.6%) | 8 (3.0%) | 12 (3.0%) | 18 (4.4%) | 0.944 | 105 (3.6%) |
| GA | 552 (29.8%) | 82 (31.1%) | 125 (31.5%) | 124 (30.2%) |  | 883 (30.2%) |
| GG | 1231 (66.5%) | 174 (65.9%) | 260 (65.5%) | 269 (65.5%) |  | 1934 (66.2%) |
| **rs985695** |  |  |  |  |  |  |
| CC | 1325 (71.7%) | 176 (66.7%) | 293 (74.0%) | 301 (73.2%) | 0.458 | 2095 (71.8%) |
| CT | 477 (25.8%) | 80 (30.3%) | 93 (23.5%) | 103 (25.1%) |  | 753 (25.8%) |
| TT | 46 (2.5%) | 8 (3.0%) | 10 (2.5%) | 7 (1.7%) |  | 71 (2.4%) |
| **rs2179922** |  |  |  |  |  |  |
| AA | 27 (1.5%) | 3 (1.1%) | 3 (0.8%) | 9 (2.2%) | 0.742 | 42 (1.4%) |
| GA | 364 (19.7%) | 50 (18.9%) | 84 (21.2%) | 78 (19.1%) |  | 576 (19.8%) |
| GG | 1454 (78.8%) | 211 (79.9%) | 310 (78.1%) | 322 (78.7%) |  | 2297 (78.8%) |
| **rs726281** |  |  |  |  |  |  |
| AA | 973 (52.6%) | 132 (50.0%) | 218 (54.9%) | 208 (50.6%) | 0.821 | 1531 (52.4%) |
| AG | 717 (38.8%) | 108 (40.9%) | 151 (38.0%) | 167 (40.6%) |  | 1143 (39.1%) |
| GG | 160 (8.6%) | 24 (9.1%) | 28 (7.1%) | 36 (8.8%) |  | 248 (8.5%) |
| **rs13216134** |  |  |  |  |  |  |
| AA | 1385 (74.9%) | 193 (73.1%) | 312 (78.6%) | 305 (74.2%) | 0.444 | 2195 (75.1%) |
| AG | 429 (23.2%) | 67 (25.4%) | 78 (19.6%) | 102 (24.8%) |  | 676 (23.1%) |
| GG | 36 (1.9%) | 4 (1.5%) | 7 (1.8%) | 4 (1.0%) |  | 51 (1.7%) |
| **rs3020418** |  |  |  |  |  |  |
| AA | 194 (10.5%) | 29 (11.0%) | 37 (9.3%) | 52 (12.7%) | 0.622 | 312 (10.7%) |
| GA | 806 (43.6%) | 108 (40.9%) | 165 (41.6%) | 165 (40.1%) |  | 1244 (42.6%) |
| GG | 848 (45.9%) | 127 (48.1%) | 195 (49.1%) | 194 (47.2%) |  | 1364 (46.7%) |
| **rs2982712** |  |  |  |  |  |  |
| CC | 433 (23.4%) | 71 (26.9%) | 91 (22.9%) | 103 (25.1%) | 0.222 | 698 (23.9%) |
| TC | 945 (51.1%) | 120 (45.5%) | 195 (49.1%) | 184 (44.8%) |  | 1444 (49.4%) |
| TT | 471 (25.5%) | 73 (27.7%) | 111 (28.0%) | 124 (30.2%) |  | 779 (26.7%) |
| **rs2273207** |  |  |  |  |  |  |
| AA | 1454 (78.6%) | 206 (78.0%) | 321 (80.9%) | 325 (79.1%) | 0.812 | 2306 (78.9%) |
| AG | 372 (20.1%) | 57 (21.6%) | 71 (17.9%) | 82 (20.0%) |  | 582 (19.9%) |
| GG | 24 (1.3%) | 1 (0.4%) | 5 (1.3%) | 4 (1.0%) |  | 34 (1.2%) |
| **rs2207396** |  |  |  |  |  |  |
| AA | 103 (5.6%) | 15 (5.7%) | 30 (7.6%) | 34 (8.3%) | 0.211 | 182 (6.2%) |
| GA | 692 (37.4%) | 94 (35.6%) | 130 (32.7%) | 140 (34.1%) |  | 1056 (36.1%) |
| GG | 1055 (57.0%) | 155 (58.7%) | 237 (59.7%) | 237 (57.7%) |  | 1684 (57.6%) |
| **rs974276** |  |  |  |  |  |  |
| AA | 1371 (74.2%) | 191 (72.9%) | 305 (76.8%) | 302 (73.7%) | 0.802 | 2169 (74.4%) |
| AG | 438 (23.7%) | 66 (25.2%) | 85 (21.4%) | 103 (25.1%) |  | 692 (23.7%) |
| GG | 38 (2.1%) | 5 (1.9%) | 7 (1.8%) | 5 (1.2%) |  | 55 (1.9%) |
| **rs9341019** |  |  |  |  |  |  |
| AA | 1579 (85.4%) | 222 (84.4%) | 339 (85.4%) | 359 (87.3%) | 0.335 | 2499 (85.6%) |
| AC | 255 (13.8%) | 37 (14.1%) | 54 (13.6%) | 52 (12.7%) |  | 398 (13.6%) |
| CC | 16 (0.9%) | 4 (1.5%) | 4 (1.0%) | 0 (0%) |  | 24 (0.8%) |
| **rs9341066** |  |  |  |  |  |  |
| AA | 5 (0.3%) | 2 (0.9%) | 1 (0.3%) | 2 (0.6%) | 0.709 | 10 (0.4%) |
| GA | 162 (9.2%) | 19 (8.1%) | 34 (10.1%) | 32 (9.1%) |  | 247 (9.2%) |
| GG | 1596 (90.5%) | 214 (91.1%) | 303 (89.6%) | 319 (90.4%) |  | 2432 (90.4%) |
| **rs2228480** |  |  |  |  |  |  |
| AA | 66 (3.6%) | 11 (4.2%) | 14 (3.5%) | 10 (2.4%) | 0.398 | 101 (3.5%) |
| GA | 571 (30.9%) | 81 (30.7%) | 109 (27.5%) | 110 (26.8%) |  | 871 (29.8%) |
| GG | 1213 (65.6%) | 172 (65.2%) | 274 (69.0%) | 291 (70.8%) |  | 1950 (66.7%) |
| **rs4986938** |  |  |  |  |  |  |
| CC | 764 (41.3%) | 108 (41.1%) | 174 (43.8%) | 179 (43.6%) | 0.686 | 1225 (42.0%) |
| CT | 851 (46.0%) | 123 (46.8%) | 165 (41.6%) | 184 (44.8%) |  | 1323 (45.3%) |
| TT | 234 (12.7%) | 32 (12.2%) | 58 (14.6%) | 48 (11.7%) |  | 372 (12.7%) |
| **rs944050** |  |  |  |  |  |  |
| CC | 6 (0.3%) | 0 (0%) | 0 (0%) | 1 (0.3%) | 0.952 | 7 (0.3%) |
| TC | 176 (10.0%) | 19 (8.1%) | 33 (9.8%) | 35 (9.9%) |  | 263 (9.8%) |
| TT | 1583 (89.7%) | 217 (91.9%) | 305 (90.2%) | 317 (89.8%) |  | 2422 (90.0%) |
| **rs1256061** |  |  |  |  |  |  |
| GG | 486 (26.3%) | 74 (28.0%) | 111 (28.0%) | 107 (26.0%) | 0.654 | 778 (26.6%) |
| GT | 940 (50.9%) | 125 (47.3%) | 208 (52.4%) | 218 (53.0%) |  | 1491 (51.1%) |
| TT | 422 (22.8%) | 65 (24.6%) | 78 (19.6%) | 86 (20.9%) |  | 651 (22.3%) |
| **rs1256059** |  |  |  |  |  |  |
| AA | 372 (20.1%) | 59 (22.5%) | 90 (22.7%) | 82 (20.0%) | 0.856 | 603 (20.7%) |
| GA | 934 (50.6%) | 129 (49.2%) | 200 (50.5%) | 214 (52.1%) |  | 1477 (50.7%) |
| GG | 541 (29.3%) | 74 (28.2%) | 106 (26.8%) | 115 (28.0%) |  | 836 (28.7%) |
| **rs8017441** |  |  |  |  |  |  |
| AA | 1647 (89.0%) | 240 (90.9%) | 352 (88.9%) | 364 (88.6%) | 0.711 | 2603 (89.1%) |
| AG | 196 (10.6%) | 22 (8.3%) | 43 (10.9%) | 44 (10.7%) |  | 305 (10.4%) |
| GG | 7 (0.4%) | 2 (0.8%) | 1 (0.3%) | 3 (0.7%) |  | 13 (0.4%) |
| **rs4365213** |  |  |  |  |  |  |
| CC | 341 (18.5%) | 54 (20.5%) | 72 (18.2%) | 71 (17.3%) | 0.87 | 538 (18.4%) |
| TC | 938 (50.8%) | 128 (48.5%) | 191 (48.2%) | 212 (51.6%) |  | 1469 (50.4%) |
| TT | 567 (30.7%) | 82 (31.1%) | 133 (33.6%) | 128 (31.1%) |  | 910 (31.2%) |
| **rs12435857** |  |  |  |  |  |  |
| AA | 340 (18.4%) | 53 (20.1%) | 72 (18.1%) | 71 (17.3%) | 0.891 | 536 (18.3%) |
| GA | 938 (50.7%) | 129 (48.9%) | 191 (48.1%) | 212 (51.6%) |  | 1470 (50.3%) |
| GG | 571 (30.9%) | 82 (31.1%) | 134 (33.8%) | 128 (31.1%) |  | 915 (31.3%) |
| **rs1256045** |  |  |  |  |  |  |
| AA | 374 (20.3%) | 57 (21.7%) | 90 (22.7%) | 81 (19.8%) | 0.861 | 602 (20.7%) |
| CA | 927 (50.2%) | 130 (49.4%) | 200 (50.5%) | 214 (52.2%) |  | 1471 (50.5%) |
| CC | 545 (29.5%) | 76 (28.9%) | 106 (26.8%) | 115 (28.0%) |  | 842 (28.9%) |
| **rs10148269** |  |  |  |  |  |  |
| AA | 370 (20.1%) | 57 (21.6%) | 85 (21.7%) | 81 (19.8%) | 0.939 | 593 (20.4%) |
| GA | 927 (50.4%) | 130 (49.2%) | 201 (51.3%) | 213 (52.1%) |  | 1471 (50.6%) |
| GG | 543 (29.5%) | 77 (29.2%) | 106 (27.0%) | 115 (28.1%) |  | 841 (29.0%) |
| **rs1273196** |  |  |  |  |  |  |
| AA | 1537 (87.1%) | 211 (89.4%) | 297 (87.9%) | 304 (86.1%) | 0.712 | 2349 (87.3%) |
| AG | 217 (12.3%) | 24 (10.2%) | 41 (12.1%) | 46 (13.0%) |  | 328 (12.2%) |
| GG | 10 (0.6%) | 1 (0.4%) | 0 (0%) | 3 (0.8%) |  | 14 (0.5%) |
| **rs10143616** |  |  |  |  |  |  |
| AA | 410 (22.2%) | 59 (22.3%) | 89 (22.4%) | 90 (21.9%) | 0.897 | 648 (22.2%) |
| GA | 962 (52.1%) | 137 (51.9%) | 193 (48.6%) | 209 (50.9%) |  | 1501 (51.4%) |
| GG | 476 (25.8%) | 68 (25.8%) | 115 (29.0%) | 112 (27.3%) |  | 771 (26.4%) |
| **rs1256031** |  |  |  |  |  |  |
| AA | 502 (27.1%) | 70 (26.5%) | 101 (25.4%) | 102 (24.8%) | 0.844 | 775 (26.5%) |
| AG | 946 (51.2%) | 133 (50.4%) | 198 (49.9%) | 216 (52.6%) |  | 1493 (51.1%) |
| GG | 401 (21.7%) | 61 (23.1%) | 98 (24.7%) | 93 (22.6%) |  | 653 (22.4%) |
| **rs17179740** |  |  |  |  |  |  |
| AA | 266 (14.4%) | 29 (11.0%) | 59 (14.9%) | 52 (12.7%) | 0.509 | 406 (13.9%) |
| GA | 875 (47.3%) | 136 (51.5%) | 178 (44.8%) | 190 (46.2%) |  | 1379 (47.2%) |
| GG | 709 (38.3%) | 99 (37.5%) | 160 (40.3%) | 169 (41.1%) |  | 1137 (38.9%) |
